# Relationship between response to aripiprazole once-monthly and paliperidone palmitate on work readiness and functioning in schizophrenia: A post-hoc analysis of the QUALIFY study

**DOI:** 10.1371/journal.pone.0183475

**Published:** 2017-08-24

**Authors:** Steven G. Potkin, Jean-Yves Loze, Carlos Forray, Ross A. Baker, Christophe Sapin, Timothy Peters-Strickland, Maud Beillat, Anna-Greta Nylander, Peter Hertel, Simon Nitschky Schmidt, Anders Ettrup, Anna Eramo, Karina Hansen, Dieter Naber

**Affiliations:** 1 Department of Psychiatry and Human Behavior, University of California, Irvine, California, United States of America; 2 Otsuka Pharmaceutical Europe Limited., Wexham, United Kingdom; 3 Lundbeck LLC, Paramus, New Jersey, United States of America; 4 Otsuka Pharmaceutical Development & Commercialization, Inc., Princeton, New Jersey, United States of America; 5 Lundbeck SAS, Issy-les-Moulineaux, France; 6 H. Lundbeck A/S, Valby, Denmark; 7 Lundbeck LLC, Deerfield, Illinois, United States of America; 8 Department of Psychiatry and Psychotherapy, University Medical Center Hamburg-Eppendorf, Hamburg, Germany; University of California San Francisco School of Medicine, UNITED STATES

## Abstract

Schizophrenia is a chronic disease with negative impact on patients’ employment status and quality of life. This post-hoc analysis uses data from the QUALIFY study to elucidate the relationship between work readiness and health-related quality of life and functioning. QUALIFY was a 28-week, randomized study (NCT01795547) comparing the treatment effectiveness of aripiprazole once-monthly 400 mg and paliperidone palmitate once-monthly using the Heinrichs-Carpenter Quality-of-Life Scale as the primary endpoint. Also, patients’ capacity to work and work readiness (Yes/No) was assessed with the Work Readiness Questionnaire. We categorized patients, irrespective of treatment, by work readiness at baseline and week 28: No to Yes (n = 41), Yes to Yes (n = 49), or No at week 28 (n = 118). Quality-of-Life Scale total, domains, and item scores were assessed with a mixed model of repeated measures. Patients who shifted from No to Yes in work readiness showed robust improvements on Quality-of-Life Scale total scores, significantly greater than patients not ready to work at week 28 (least squares mean difference: 11.6±2.6, p<0.0001). Scores on Quality-of-Life Scale instrumental role domain and items therein–occupational role, work functioning, work levels, work satisfaction–significantly improved in patients shifting from No to Yes in work readiness (vs patients No at Week 28). Quality-of-Life Scale total scores also significantly predicted work readiness at week 28. Overall, these results highlight a strong association between improvements in health-related quality of life and work readiness, and suggest that increasing patients’ capacity to work is an achievable and meaningful goal in the treatment of impaired functioning in schizophrenia.

## Introduction

Schizophrenia is a chronic and disabling disease in which repeated relapses have a negative impact on patients’ functioning and are disruptive for patients’ education and employment [1]. Unemployment is a large burden for patients as well as for society, and on average, the worldwide proportion of patients with schizophrenia who are not undertaking any paid employment is around 80% [2]. Unemployment has socioeconomic consequences for the patient’s ability to live independently and participate actively in the community and thus can adversely impact quality of life [3]. Improved quality of life and functioning is an essential long-term treatment goal in schizophrenia for patients, caregivers, clinicians, and payers that is receiving increasing scientific and societal attention [4, 5]. Therefore, evaluating the ability to function within a working environment (i.e. work readiness) in schizophrenia is of clinical relevance, and increasing the functional capacity to work should be considered a valuable goal in the treatment of schizophrenia, increasing patients’ social integration in the community. Systematic assessments of capacity to work and work readiness, such as the clinician-rated Readiness for Work Questionnaire (WoRQ), constitute a practical and validated approach to assess treatment-induced improvement of functioning in patients with schizophrenia [6]. The simplicity of the Yes/No question as to whether the patient is ready to work is potentially useful in clinical practice, where more lengthy, detailed scales are unlikely to be administered routinely.

The QUALIFY (QUAlity of LIfe with AbiliFY Maintena^®^) study is one of the few randomized studies directly comparing two different long-acting injectable antipsychotics (LAIs), and was the first to compare the effects of two atypical LAIs on a measure of health-related quality of life and functioning as the primary outcome. The Heinrichs-Carpenter Quality-of-Life scale (QLS) is a detailed assessment [7], broadly defining functioning as an individual’s ability to perform normal daily activities required to meet basic needs, fulfil usual roles, and maintain their health and well-being [8]. The QLS is one of the relatively few rating scales designed to assess aspects of functional impairment (social, occupational, and psychological) associated with schizophrenia, and it is sensitive to subtle change over time, as well as to the psychopharmacological effect of treatment. Thus, QLS is the most widely used instrument for assessing health-related quality of life in schizophrenia [9]. A drawback of the clinician-rated QLS is that the long administration time (30–45 minutes) can be burdensome for the patient as well as the clinician [10].

The primary analysis of the QUALIFY study, performed using a mixed model for repeated measures (MMRM), showed non-inferior and superior improvements with AOM 400 vs PP on QLS total score over 28 weeks [11]. Furthermore, the QUALIFY study was the first study to apply the WoRQ instrument to assess differences between two treatments for schizophrenia. The effect of AOM 400 and PP on work readiness was analyzed post-hoc using logistic regression adjusted for baseline status to compare odds of work readiness after 28 weeks of treatment. We recently reported significantly greater improvement on WoRQ total scores with AOM 400 vs PP, as well as significantly more patients improving in work readiness status after AOM 400 vs PP treatment [12].

As a randomized phase IV study, QUALIFY is a rich data source enabling cross-validation of instruments used in the evaluation of functioning in schizophrenia. This study is particularly well-suited to investigate the association between improvements on different scales of health-related quality of life and functioning after switching to LAI therapy.

In this post-hoc analysis of the QUALIFY study, we investigated the association between response to LAI treatment with AOM 400 or PP on measures of patient functioning and work readiness (QLS and WoRQ).

## Methods

### Study design

This was a post-hoc analysis of data derived from the QUALIFY study, a randomized controlled trial comparing the atypical LAIs, AOM 400 and PP in stable adult patients, ages 18 to 60 years, with schizophrenia (defined by DSM-IV-TR). The study design and patient population of the QUALIFY study were previously described in detail [11]; the protocol was approved by the relevant institutional review board for each country in which the trial was conducted (S1 Text), all patients provided written informed consent, and the study was conducted in accordance with the Declaration of Helsinki. Briefly, included patients were switching from oral to LAI antipsychotic treatment and had clinical global impression—severity (CGI-S) scores ≥3 (mildly ill) and ≤5 (markedly ill) at the screening and baseline visits. Patients with a diagnosis of psychiatric disorder or DSM-IV-TR axis I disorder other than schizophrenia or acute exacerbation of psychotic symptoms, or hospitalization for >3 months before the screening visit were excluded. This post-hoc analysis was longitudinal and observational in nature, and combined all patients in the QUALIFY study, regardless of treatment group, to determine the effect of treatment response on patient functioning and work readiness.

### Assessments

The QLS (Table 1) was the primary outcome of the QUALIFY study [11], assessed at baseline and at weeks 4, 8, 16, and 28 (end of study) by a rater blinded to the treatment. The QLS comprises 21 items in 4 domains [7]. In addition, a number of secondary outcomes were evaluated in the QUALIFY study, including WoRQ (Table 1) which was rated at baseline and at week 28 (end of study). WoRQ was administered by a rater who was not blinded to the treatment (a different rater from the one assessing QLS).

**Table 1:** Overview of the Heinrichs-Carpenter Quality of Life Scale (QLS) and Work Readiness Questionnaire (WoRQ).

	**QLS**				**WoRQ**
**Objective**	To assess health-related quality of life and functioning in patients with schizophrenia during the preceding four weeks [7]				To assess functional capacity to work and work readiness in patients with schizophrenia [6]
**Rater**	Clinician				Clinician
**Domain**	Interpersonal relations	Instrumental role	Intrapsychic foundations^a^	Common objects and activities	
**Purpose**	To examine a patient’s social experience	To assess a patient’s work functioning	To assess a patient’s sense of purpose and motivation	To evaluate a patient’s level of participation in the community	
**Items**	1. Household^a^ 2. Friends 3. Acquaintances 4. Social activity 5. Social network 6. Social initiative 7. Withdrawal 8. Sociosexual	9. Occupational role 10. Work functioning 11. Work level 12. Work satisfaction^a^	13. Sense of purpose 14. Motivation 15. Curiosity 16. Anhedonia 17. Aimless inactivity 20. Empathy 21. Emotional interaction	18. Commonplace objects 19. Commonplace activities	1. The patient generally adheres to a treatment plan, including medication. 2. The patient is able to carry out Activities of Daily Living. 3. The patient is able to consistently keep appointments and schedules with only minimal assistance. 4. The patient would have adequate impulse control when interacting with authority figures, peers or coworkers, and potential customers. 5. The patient’s behavior would not make others uncomfortable in a work situation. 6. The patient’s appearance would not make others uncomfortable in a work situation. 7. The patient’s current symptoms would not interfere with the ability to hold a job. 8. Based on your clinical judgment, is this patient ready for work?
**Scoring**	Each item is rated on a 7-point scale, ranging from 0 (severe impairment) to 6 (normal or unimpaired functioning), and definitions are provided for 4 anchor points of the 7 points. Higher scores indicate a better quality of life and functioning, and QLS total scores thus ranges from 0–126.				Each statement item is rated on a 4-point scale, ranging from 1 (strongly agree) to 4 (strongly disagree). In the final item 8, the clinician indicates if the patient is ready for work independent of the score in each item (Yes/No). WoRQ total score is the sum of items 1–7, and thus ranges from 7–28 with lower scores indicating better functioning.
	**QLS**				**WoRQ**
**Objective**	To assess health-related quality of life and functioning in patients with schizophrenia during the preceding four weeks^7^				To assess functional capacity to work and work readiness in patients with schizophrenia^6^
**Rater**	Clinician				Clinician
**Domain**	Interpersonal relations	Instrumental role	Intrapsychic foundations^a^	Common objects and activities	
**Purpose**	To examine a patient’s social experience	To assess a patient’s work functioning	To assess a patient’s sense of purpose and motivation	To evaluate a patient’s level of participation in the community	
**Items**	1. Household^a^ 2. Friends 3. Acquaintances 4. Social activity 5. Social network 6. Social initiative 7. Withdrawal 8. Sociosexual	9. Occupational role 10. Work functioning 11. Work level 12. Work satisfaction^a^	13. Sense of purpose 14. Motivation 15. Curiosity 16. Anhedonia 17. Aimless inactivity 20. Empathy 21. Emotional interaction	18. Commonplace objects 19. Commonplace activities	1. The patient generally adheres to a treatment plan, including medication. 2. The patient is able to carry out Activities of Daily Living. 3. The patient is able to consistently keep appointments and schedules with only minimal assistance. 4. The patient would have adequate impulse control when interacting with authority figures, peers or coworkers, and potential customers. 5. The patient’s behavior would not make others uncomfortable in a work situation. 6. The patient’s appearance would not make others uncomfortable in a work situation. 7. The patient’s current symptoms would not interfere with the ability to hold a job. 8. Based on your clinical judgment, is this patient ready for work?
**Scoring**	Each item is rated on a 7-point scale, ranging from 0 (severe impairment) to 6 (normal or unimpaired functioning), and definitions are provided for 4 anchor points of the 7 points. Higher scores indicate a better quality of life and functioning, and QLS total scores thus ranges from 0–126.				Each statement item is rated on a 4-point scale, ranging from 1 (strongly agree) to 4 (strongly disagree). In the final item 8, the clinician indicates if the patient is ready for work independent of the score in each item (Yes/No). WoRQ total score is the sum of items 1–7, and thus ranges from 7–28 with lower scores indicating better functioning.

^a^ For domain scores and total scores, scores for patients who could not be rated on items 1 and 12 were prorated on the basis on the items 2–8 and items 9–11, respectively.

The CGI-S scale [13] quantifies the clinician’s impression of the patient’s current illness severity on a scale ranging from 1 (normal, not at all ill), to 7 (among the most extremely ill patients). In the QUALIFY study, CGI-S (included as secondary endpoint) was used to assess symptom severity at all visits, namely screening, baseline and post-baseline at weeks 2, 3, 4, 8, 12, 16, 20, 24, and 28 (end of study).

### Statistical analysis

All analyses were conducted in the full analysis set, which is a modified intent-to-treat set comprising treated patients with valid baseline QLS and WoRQ assessments, at least one valid post-baseline QLS assessment, and a valid week-28 WoRQ assessment. Thus, analyses were conducted in a subset of patients from the QUALIFY study. It should be noted that the final population included in the current post-hoc analysis (n = 208) comprised those who completed the original QUALIFY study (n = 183) as well as 25 additional patients who discontinued before completing the study but did complete a week-28 WoRQ assessment.

Irrespective of AOM 400 or PP treatment, patients were categorized based on work readiness (Yes or No) at baseline and week 28: shift from No to Yes (n = 41), Yes to Yes (n = 49), or No at Week 28 (n = 118; comprising No to No patients [n = 102] and shift from Yes to No patients [n = 16]), see Table 2, item 8. Due to the low number of patients shifting in work readiness from Yes at baseline to No at week 28 (n = 7 for AOM 400; n = 9 for PP), these patients were grouped with those not ready to work either at baseline or at week 28 in the “No at Week 28”-group.

**Table 2:** Patient demographics and baseline characteristics in patients categorized by shifts from baseline to week 28 in work readiness (WoRQ item 8).

	No at Week 28	No to Yes	Yes to Yes
**Patients in the full analysis set, n**	118	41	49
**AOM 400 treatment group**	52	29	29
**PP treatment group**	66	12	20
**Baseline demographics:**			
**Age, mean (SD), years**	44.5 (10.4)	41.5 (10.9)	40.4 (11.0)
**Gender male, n (%)**	72 (61.0)	21 (51.2)	29 (59.2)
**Race, white n (%)**	85 (72.0)	23 (56.1)	40 (81.6)
**Weight, mean (SD), kg**	87.8 (18.4)	82.6 (16.8)	88.1 (20.4)
**Baseline effectiveness scores:**			
**QLS total score, mean (SD)**	57.0 (18.5)	64.8 (20.6)	81.4 (18.2)
**QLS domain scores Common objects and activities score, mean (SD)**	7.0 (2.3)	7.5 (2.0)	8.9 (2.1)
**QLS domain scores intrapsychic foundations score, mean (SD)**	20.3 (7.0)	24.0 (7.3)	28.8 (6.3)
**QLS domain scores interpersonal relations score, mean (SD)**	21.2 (8.5)	22.7 (10.0)	28.8 (9.7)
**QLS domain scores instrumental role score, mean (SD)**	8.5 (5.5)	10.6 (5.7)	15.0 (5.6)
**WoRQ total score, mean (SD)**	15.6 (2.9)	15.5 (2.8)	11.8 (2.8)
**CGI-S score, mean (SD)**	4.08 (0.63)	4.05 (0.63)	3.80 (0.61)

All baseline measures are summarized for the full analysis set. AOM 400: Aripiprazole once-monthly, CGI-S: Clinical Global Impression—Severity scale (CGI-S), PP: Paliperidone Palmitate, QLS: Heinrichs-Carpenter Quality of Life Scale, SD: Standard deviation, WoRQ: Work Readiness Questionnaire.

The changes in QLS total, domain, and item scores were compared in work readiness shift groups using a MMRM with an unstructured covariance matrix including baseline score-by-visit interaction, geographic region (Europe/North America), age group (≤35/>35 years), visit, and treatment-by-visit interaction as fixed effects. This post-hoc analysis used MMRM methodology similar to what was used in the primary analysis [11].

The time-dependence of the associations between work readiness status and QLS total scores or CGI-S scores were analyzed with logistic regressions applied to assess the predictive effects of absolute scores of QLS total and CGI-S at individual visits on the positive outcome (“Yes”) in work readiness at week 28. The logistic regressions used baseline status of readiness to work and QLS total or CGI-S scores as covariates, and input scores (QLS total or CGI-S) were standardized using mean and standard deviation for each visit to account for the numerical differences in the score ranges. As a consequence of standardization, the size of parameter estimates for QLS total and CGI-S is comparable between scales and indicates the strength of the effect of the particular score at the given visit in predicting the work readiness outcome at week 28. The positive or negative parameter estimates indicate the direction of improvement on each scale (clinical improvements correspond to higher QLS total and lower CGI-S scores), and the parameter estimates were tested for differences from zero. For the exploratory analyses presented here, p-values were considered nominal and were not corrected for multiple comparisons.

## Results

Patients who were rated as ready to work at baseline and week 28 generally showed baseline scores corresponding to higher functioning (QLS [total, and all four domains] and WoRQ) and milder disease severity (CGI-S) as compared to patients rated not ready to work at baseline (Table 2 and S1 Table).

**Fig 1:**
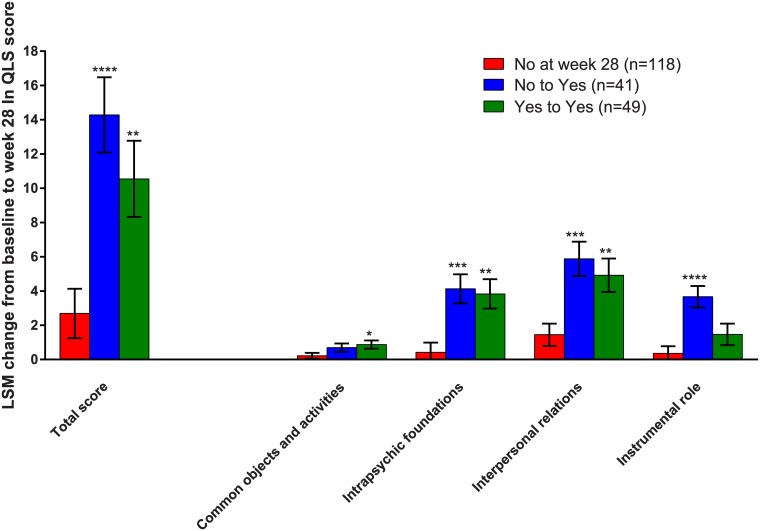
Improvements in QLS total and QLS domains by shifts in work readiness. Changes in QLS total and QLS domain scores at week 28 categorized by shifts from baseline to week 28 in work readiness (WoRQ item 8) in all patients. Least squares mean (LSM) changes from baseline to week 28 are analyzed with MMRM in the full analysis set (FAS). *p<0.05, and **p<0.01, ***p<0.001, and ****p<0.0001 indicate significant differences vs the patients rated not ready to work at week 28. Error bars indicate standard error (SE) of the LSM.

Patients who shifted from No to Yes in work readiness showed least squares mean (LSM) change from baseline to week 28 (±SE) on QLS total scores of 14.3±2.2 (Fig 1). This change was significantly greater than in the group of patients who were No at week 28 in work readiness (LSM change from baseline to week 28: 2.7±1.4; LSM difference: 11.6±2.6, 95%CI: [6.5; 16.7], p<0.0001). Patients with Yes in work readiness both at baseline and week 28 also showed significantly greater LSM changes on QLS total scores (10.5±2.2) compared with patients who were No at week 28; LSM differences: 7.9±2.7, 95%CI: [2.5; 13.2], p = 0.0045 (Fig 1).

In similar analysis as for QLS total scores, patients who were ready to work at week 28 (either No to Yes or Yes to Yes) also had significantly greater improvements relative to patients who were not ready to work at week 28, across all QLS domains. QLS instrumental role domain scores were significantly improved in patients shifting from No to Yes in work readiness compared to patients who were No at week 28; LSM difference: 3.3±0.7, 95%CI: [1.8;4.8], p<0.0001 (Fig 1). In contrast, patients judged as ready to work both at baseline and at week 28 (Yes to Yes) did not show greater improvement on QLS instrumental role domain scores compared to patients who were No at week 28 in work readiness; LSM difference: 1.1±0.8, 95%CI: [-0.4; 2.6], p = 0.150 (Fig 1).

**Fig 2:**
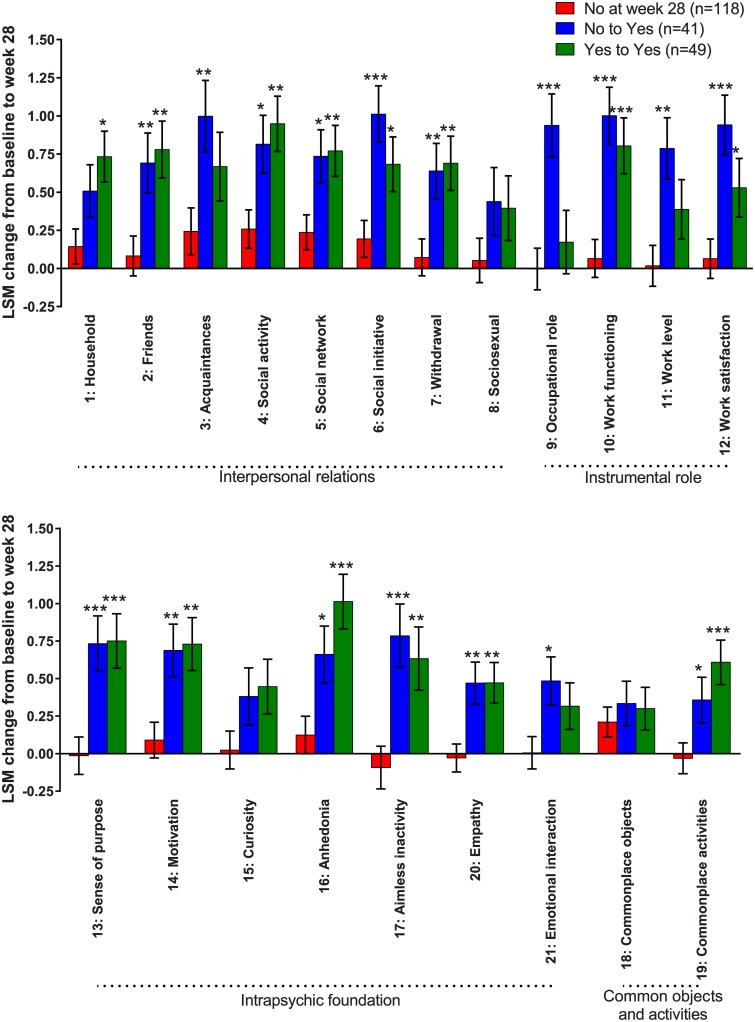
Improvements in QLS items by shifts in work readiness. Changes in QLS item scores at week 28 categorized by shifts from baseline to week 28 in work readiness (WoRQ item 8) in all patients. Least squares mean (LSM) changes from baseline to week 28 are analyzed with MMRM in the full analysis set (FAS). *p<0.05 and **p<0.01, and ***p<0.001 indicate differences vs the patients rated not ready to work at week 28. Error bars indicate standard error (SE) of the LSM.

Across QLS items, patients rated ready to work at week 28 generally showed significant improvement vs patients not ready to work at week 28 (Fig 2). On the specific items of QLS instrumental role (Items 9–12: occupational role, work functioning, work levels, work satisfaction), improvements of nearly 1 point on each item were found in the patients shifting from No to Yes in work readiness (Fig 2). In contrast, improvements on QLS items of work level and occupational role were not significantly different between patients Yes in work readiness both at baseline and week 28 versus patients who No at week 28 (Fig 2, Items 9 and 11).

**Fig 3:**
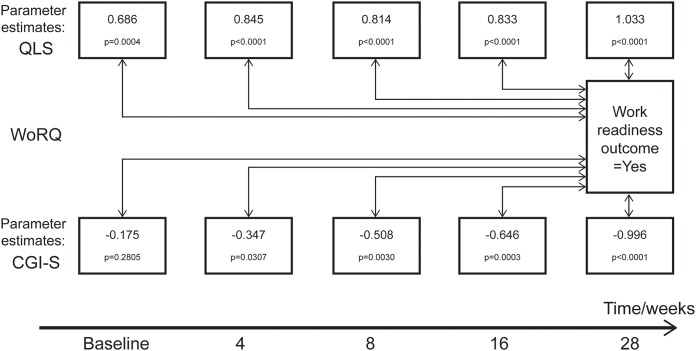
Predictability of QLS total and CGI-S scores on work readiness. Parameter estimates of the effects of QLS or CGI-S scores at the visits shown (univariate models) on work readiness at week 28 (outcome = yes). Standardized values of QLS and CGI-S absolute scores were used with baseline status of readiness to work as covariate.

The logistic regression describes how QLS total and CGI-S scores predicted readiness to work at week 28 (Fig 3). Significant associations between QLS total scores and work readiness at week 28 occurred at all visits. CGI-S scores at baseline did not significantly predict work readiness at week 28 (p = 0.2805), although CGI-S scores at subsequent visits were predictive (p≤0.0307). Furthermore, parameter estimates were generally higher when using QLS total scores as predictors (absolute values 0.686 to 1.033) as compared to CGI-S scores (absolute values 0.175 to 0.996). This suggests a closer association to work readiness, with faster onset between measured improvements, for QLS as compared to CGI-S. The parameter estimates for the predictive effect of QLS total and CGI-S scores generally increased during the study, indicating better predictions of work readiness when scores were measured closer in time to the outcome at the end of the study. The increase in the parameter estimates during the study was observed particularly for the CGI-S scores (Fig 3).

## Discussion

This post-hoc analysis of the QUALIFY study showed that after switching to atypical LAI therapy strong associations between improvement in functioning and work readiness were revealed, as measured with QLS and WoRQ, respectively. Robust and clinically relevant 14-point improvements from baseline to week 28 on QLS total score were observed in patients who concurrently shifted from No to Yes in work readiness. The 14-point improvement in QLS total score corresponds to more than twice the minimal clinically important difference [14]. The association between the QLS domain of instrumental role function and work readiness was particularly noteworthy. Furthermore, QLS total scores better predicted outcome on work readiness than did the measure of symptom severity (CGI-S). This finding demonstrates that quality of life and functional improvement can lead to a tangible and meaningful outcome for the patient (i.e., the ability to work). Overall, these results suggest that improving work readiness is achievable in patients with schizophrenia, and thus can be an important goal of treatment.

The QUALIFY study was the first to assess improvements in health-related qualify of life with concurrent systematic assessments of functional capacity to work and the data from this study provide a unique opportunity to investigate the association between treatment-related improvements in two distinct measures of functioning in schizophrenia. The patients who shifted from No to Yes in work readiness showed the largest changes on the QLS domain related to work—Instrumental role items on work role, level, performance, and satisfaction—and it is not surprising that the association between improvement in QLS and work readiness was strongest for QLS items related to work function. These results are also in line with a published outpatient study showing that the highest correlations between hours worked and QLS were also in the Instrumental role domain [15]. Our results also indicate that the independent raters in the study (QLS-raters were blinded to treatment while the WoRQ-raters were not blinded to the treatment) assessed patient improvements similarly and highlight the consistency between these two scales.

The large and consistent improvements in QLS total scores in patients shifting from No to Yes in work readiness suggesting a strong association between health-related qualify of life (QLS) and the functional capacity to work (WoRQ) is also supported by the QLS total scores at every visit significantly predicting work readiness at week 28 in the logistic regression. Although CGI-S scores also significantly predicted work readiness, the estimates of prediction were generally lower as compared to those obtained with QLS total scores. Thus, these associations suggest that general status in functioning (QLS) strongly predicts the possibility of attaining an important functional milestone (work readiness) in the treatment of schizophrenia. With regard to the CGI-S analysis, however, it should be noted that the study inclusion criteria restricted CGI-S scores to between 3 and 5. The limited range allowed for baseline CGI-S may have affected the parameter estimate of predictability and resulted in the absence of a significant association between CGI-S scores at baseline and work readiness at week 28. Nevertheless, the association between baseline CGI-S and work readiness at week 28 strengthened during the study, as illustrated by increases in parameter estimates for CGI-S scores at each subsequent post-baseline visit. Despite the time-dependent increases in CGI-S predictability, larger parameter estimates at all visits for QLS still suggest stronger predictive value of QLS total scores than for CGI-S scores on improvements in work readiness.

The QUALIFY study used CGI-S as a surrogate measurement of clinical status, instead of symptom severity as measured by the Positive and Negative Syndrome Scale or the Brief Psychiatric Rating Scale. Previous studies have shown a close correlation between Positive and Negative Syndrome Scale, Brief Psychiatric Rating Scale and CGI-S [16]. These post-hoc analyses suggest that improvements in health-related quality of life and functioning have a stronger association with readiness to work when compared to the association between global clinical impression and work readiness. This is in line with a previous report demonstrating that improvements in functioning are distinct from symptom improvements and improvements in functioning and quality of life extend beyond psychopathological changes [17]. Furthermore, these recent guidelines for the treatment of schizophrenia also reinforce improvements in functioning and quality of life as main goals of treatment during the stable phase after ensuring that symptom remission or control is sustained.

It should be noted that the present post-hoc analyses are exploratory in nature. Furthermore, the analyses were done with both treatment groups combined, and thus do not serve to compare AOM 400 and PP in the treatment of schizophrenia. However, we have previously reported pre-specified analyses showing superior improvements with AOM 400 (n = 136) vs PP (n = 132) on QLS total score [11], as well as significantly greater improvements on WoRQ total scores and work readiness with AOM 400 vs PP[12].

In summary, the large and consistent improvement in mean QLS scores in patients shifting from No to Yes in work readiness suggests a strong association between health-related qualify of life (QLS) and functional capacity to work (WoRQ). These findings highlight that quality of life improvements can translate to tangible and meaningful outcomes for the patient, and importantly, suggest that increasing patients’ capacity to work may be a more realistic goal in the treatment of schizophrenia than previously realized. Evaluating capacity to work may further optimize functioning in schizophrenia by directing rehabilitation services to the subgroup with evaluated capacity to work. The WoRQ scale may be useful as a practical, brief, and systematic measure of work readiness that reflects broader functioning in patients with schizophrenia.

## Supporting information

S1 Text Institutional review boards. (DOCX)

S1 Table Change from baseline in QLS scores by shifts in readiness to work on the WoRQ scale. (DOCX)
