# Correction: Relationship between response to aripiprazole once-monthly and paliperidone palmitate on work readiness and functioning in schizophrenia: A post-hoc analysis of the QUALIFY study

**DOI:** 10.1371/journal.pone.0188416

**Published:** 2017-11-20

**Authors:** The PLOS ONE Staff

The content in Table 1 is incorrectly duplicated. The publisher apologizes for the error. Please see the correct Table 1 here.

**Table 1:** Overview of the Heinrichs-Carpenter Quality of Life Scale (QLS) and Work Readiness Questionnaire (WoRQ).

	QLS				WoRQ
**Objective**	To assess health-related quality of life and functioning in patients with schizophrenia during the preceding four weeks^7^				To assess functional capacity to work and work readiness in patients with schizophrenia^6^
**Rater**	Clinician				Clinician
**Domain**	Interpersonal relations	Instrumental role	Intrapsychic foundations^a^	Common objects and activities	
**Purpose**	To examine a patient’s social experience	To assess a patient’s work functioning	To assess a patient’s sense of purpose and motivation	To evaluate a patient’s level of participation in the community	
**Items**	1. Household^a^ 2. Friends 3. Acquaintances 4. Social activity 5. Social network 6. Social initiative 7. Withdrawal 8. Sociosexual	9. Occupational role 10. Work functioning 11. Work level 12. Work satisfaction^a^	13. Sense of purpose 14. Motivation 15. Curiosity 16. Anhedonia 17. Aimless inactivity 20. Empathy 21. Emotional interaction	18. Commonplace objects 19. Commonplace activities	1. The patient generally adheres to a treatment plan, including medication. 2. The patient is able to carry out Activities of Daily Living. 3. The patient is able to consistently keep appointments and schedules with only minimal assistance. 4. The patient would have adequate impulse control when interacting with authority figures, peers or coworkers, and potential customers. 5. The patient’s behavior would not make others uncomfortable in a work situation. 6. The patient’s appearance would not make others uncomfortable in a work situation. 7. The patient’s current symptoms would not interfere with the ability to hold a job. 8. Based on your clinical judgment, is this patient ready for work?
**Scoring**	Each item is rated on a 7-point scale, ranging from 0 (severe impairment) to 6 (normal or unimpaired functioning), and definitions are provided for 4 anchor points of the 7 points. Higher scores indicate a better quality of life and functioning, and QLS total scores thus ranges from 0–126.				Each statement item is rated on a 4-point scale, ranging from 1 (strongly agree) to 4 (strongly disagree). In the final item 8, the clinician indicates if the patient is ready for work independent of the score in each item (Yes/No). WoRQ total score is the sum of items 1–7, and thus ranges from 7–28 with lower scores indicating better functioning.

^a^ For domain scores and total scores, scores for patients who could not be rated on items 1 and 12 were prorated on the basis on the items 2–8 and items 9–11, respectively.
