# Supplementary material for: Relationship between response to aripiprazole once-monthly and paliperidone palmitate on work readiness and functioning in schizophrenia: A post-hoc analysis of the QUALIFY study
Source: PLoS One. 2017 Aug 24;12(8):e0183475. doi: 10.1371/journal.pone.0183475 (PMC5570322; doi:10.1371/journal.pone.0183475)
Supplement: S1 Text — (DOCX) [file pone.0183475.s001.docx]

**Appendix**

Institutional Review Boards

Canada: Copernicus Group IRB, Aurora, Ontario; Queen's University & Affiliated Teaching Hospitals Research Ethics Board, Kingston, Ontario; Comite d'ethique de la recherche de l'Hopital Louis-H. Lafontaine Montreal, Quebec; Comité d'éthique de la recherche de l'Institut Universitair en santé mentale de Quebec, Quebec; Windsor Regional Hospital Research Ethics Board Windsor, Ontario; University of Calgary, Calgary, Ontario.

Czech Republic: Eticka komise FN Brno, Brno.

Estonia: Research Ethics Committee of the University of Tartu, Tartu.

France: Comité de Protection des Personnes Ouest V, Rennes cedex, Seine St Denis.

Germany: Landesamt fuer Gesundheit und Soziales Berlin, Berlin.

Italy: Comitato Etico della ASL Cagliari, Cagliari; Comitato Etico Indipendente Presso la Fondazione PTV Policlinico, Rome; Comitato Etico per la sperimentazione clinica della Provincia di Treviso CEP TV; Comitato Etico dell'Azienda Ospedaliera Universitaria Maggiore della Carità di Novara; Comitato Etico della Azienda Sanitaria Locale BAT - DSS 2 Andria; Comitato Etico dell'Azienda Ospedaliera Spedali Civili di Brescia; Comitato Etico dell'Azienda Ospedaliero Universiitaria S.Martino di Genova; Comitato Etico dell'Azienda Ospedaliera S. Gerardo di Monza (MB); Comitato Etico dell'Azienda Ospedaliera Policlinico Consorziale di Bari; Comitato Etico per la Sperimentazione dell'Azienda Ospedaliera di Padova; Comitato Etico dell'Azienda Ospedaliero - Universitaria Policlinico Vittorio Emanuele di Catania; Comitato Etico Locale per la Sperim. Clin. dei Medicinali dell'Az. Osp.ra Univ.ria Senese di Siena; Comitato Etico Locale per la Sperimentazione Clinica della AUSL 12 di Viareggio; Comitato Etico dell'Azienda Ospedaliera S. Andrea di Roma.

Spain: CEIC Hospital Clinic, Barcelona.

Sweden: Regionala etikprövningsnämnden i Stockholm, Stockholm.

United Kingdom: NRES Committee West of Scotland REC 1, Glasgow, Strathclyde.

United States: Copernicus Group IRB, Research Triangle Park, North Carolina; Washington University in St. Louis IRB, St. Louis, Missouri.
