# Supplementary material for: Relationship between response to aripiprazole once-monthly and paliperidone palmitate on work readiness and functioning in schizophrenia: A post-hoc analysis of the QUALIFY study
Source: PLoS One. 2017 Aug 24;12(8):e0183475. doi: 10.1371/journal.pone.0183475 (PMC5570322; doi:10.1371/journal.pone.0183475)
Supplement: S1 Table — (DOCX) [file pone.0183475.s002.docx]

| Supplemental Table. Repeated Measures Analysis of Change from Baseline in QLS Score by all items, domains and total in the 3 shift groups (Readiness to work on the WoRQ scale) (FAS Population) | | | | | | | | | | | | |
| --- | --- | --- | --- | --- | --- | --- | --- | --- | --- | --- | --- | --- |
|  | | | | | | | | **Comparison to No at week 28** | | | | |
|  | | **Baseline** | |  | | | | | | **95% CI** | |  |
| **QLS item/domain** | **Treatment**  **Group** | **N** | **Mean** | **Visit** | **N** | **Mean** | **SE** | **Diff.** | **SE** | **Lower** | **Upper** | **p-value** |
|  | | | | | | | | | | | | |
| TOTAL COMMON OBJECTS AND ACTIVITIES SCORE | No at week 28 | 118 | 6.9831 | Week 04 | 118 | -0.0297 | 0.1285 |  |  |  |  |  |
|  |  |  |  | Week 08 | 118 | 0.0428 | 0.1479 |  |  |  |  |  |
|  |  |  |  | Week 16 | 118 | 0.1805 | 0.1534 |  |  |  |  |  |
|  |  |  |  | Week 28 | 100 | 0.2169 | 0.1647 |  |  |  |  |  |
|  | | | | | | | | | | | | |
|  | No to Yes | 41 | 7.5366 | Week 04 | 41 | 0.3397 | 0.1889 | 0.3694 | 0.2207 | -0.0658 | 0.8046 | 0.0958 |
|  |  |  |  | Week 08 | 40 | 0.6486 | 0.2265 | 0.6058 | 0.2641 | 0.0850 | 1.1265 | 0.0228 |
|  |  |  |  | Week 16 | 41 | 0.5128 | 0.2351 | 0.3323 | 0.2745 | -0.2089 | 0.8735 | 0.2275 |
|  |  |  |  | Week 28 | 40 | 0.6929 | 0.2451 | 0.4760 | 0.2894 | -0.0947 | 1.0467 | 0.1016 |
|  | | | | | | | | | | | | |
|  | Yes to Yes | 49 | 8.8776 | Week 04 | 49 | 0.4778 | 0.1867 | 0.5075 | 0.2184 | 0.0769 | 0.9380 | 0.0211 |
|  |  |  |  | Week 08 | 49 | 0.6130 | 0.2203 | 0.5702 | 0.2600 | 0.0575 | 1.0828 | 0.0295 |
|  |  |  |  | Week 16 | 49 | 0.6542 | 0.2297 | 0.4737 | 0.2716 | -0.0618 | 1.0092 | 0.0826 |
|  |  |  |  | Week 28 | 47 | 0.8780 | 0.2397 | 0.6612 | 0.2881 | 0.0930 | 1.2293 | 0.0228 |
|  | | | | | | | | | | | | |
| TOTAL INTRAPSYCHIC FOUNDATIONS SCORE | No at week 28 | 118 | 20.2712 | Week 04 | 118 | 0.4637 | 0.3738 |  |  |  |  |  |
|  |  |  |  | Week 08 | 118 | 0.7440 | 0.4242 |  |  |  |  |  |
|  |  |  |  | Week 16 | 118 | 0.4585 | 0.5384 |  |  |  |  |  |
|  |  |  |  | Week 28 | 100 | 0.4189 | 0.5697 |  |  |  |  |  |
|  | | | | | | | | | | | | |
|  | No to Yes | 41 | 23.9756 | Week 04 | 41 | 1.8523 | 0.5408 | 1.3886 | 0.6405 | 0.1257 | 2.6516 | 0.0313 |
|  |  |  |  | Week 08 | 40 | 2.0228 | 0.6356 | 1.2788 | 0.7515 | -0.2029 | 2.7606 | 0.0903 |
|  |  |  |  | Week 16 | 41 | 2.9751 | 0.8306 | 2.5166 | 0.9831 | 0.5783 | 4.4550 | 0.0112 |
|  |  |  |  | Week 28 | 40 | 4.1254 | 0.8507 | 3.7065 | 1.0169 | 1.7006 | 5.7124 | 0.0003 |
|  | | | | | | | | | | | | |
|  | Yes to Yes | 49 | 28.7755 | Week 04 | 49 | 1.9603 | 0.5522 | 1.4966 | 0.6588 | 0.1974 | 2.7958 | 0.0242 |
|  |  |  |  | Week 08 | 49 | 2.9428 | 0.6392 | 2.1989 | 0.7695 | 0.6815 | 3.7162 | 0.0047 |
|  |  |  |  | Week 16 | 49 | 3.2030 | 0.8315 | 2.7445 | 1.0117 | 0.7497 | 4.7393 | 0.0072 |
|  |  |  |  | Week 28 | 47 | 3.8295 | 0.8562 | 3.4106 | 1.0526 | 1.3343 | 5.4869 | 0.0014 |
|  | | | | | | | | | | | | |
| TOTAL INTERPERSONAL RELATIONS SCORE | No at week 28 | 118 | 21.2228 | Week 04 | 118 | 0.3841 | 0.4444 |  |  |  |  |  |
|  |  |  |  | Week 08 | 118 | 1.4180 | 0.4612 |  |  |  |  |  |
|  |  |  |  | Week 16 | 118 | 1.3464 | 0.5871 |  |  |  |  |  |
|  |  |  |  | Week 28 | 100 | 1.4482 | 0.6526 |  |  |  |  |  |
|  | | | | | | | | | | | | |
|  | No to Yes | 41 | 22.7282 | Week 04 | 41 | 1.3823 | 0.6607 | 0.9983 | 0.7690 | -0.5180 | 2.5145 | 0.1957 |
|  |  |  |  | Week 08 | 40 | 2.0514 | 0.6957 | 0.6335 | 0.8092 | -0.9619 | 2.2288 | 0.4346 |
|  |  |  |  | Week 16 | 41 | 3.8729 | 0.9206 | 2.5265 | 1.0709 | 0.4151 | 4.6379 | 0.0193 |
|  |  |  |  | Week 28 | 40 | 5.8788 | 1.0005 | 4.4306 | 1.1743 | 2.1148 | 6.7464 | 0.0002 |
|  | | | | | | | | | | | | |
|  | Yes to Yes | 49 | 28.7988 | Week 04 | 49 | 2.0568 | 0.6554 | 1.6728 | 0.7597 | 0.1749 | 3.1707 | 0.0288 |
|  |  |  |  | Week 08 | 49 | 3.1555 | 0.6842 | 1.7376 | 0.7958 | 0.1684 | 3.3067 | 0.0302 |
|  |  |  |  | Week 16 | 49 | 3.9482 | 0.8960 | 2.6019 | 1.0586 | 0.5148 | 4.6889 | 0.0148 |
|  |  |  |  | Week 28 | 47 | 4.9170 | 0.9739 | 3.4688 | 1.1631 | 1.1752 | 5.7625 | 0.0032 |
|  | | | | | | | | | | | | |
| TOTAL INSTRUMENTAL ROLE SCORE | No at week 28 | 118 | 8.5395 | Week 04 | 118 | 0.3860 | 0.2756 |  |  |  |  |  |
|  |  |  |  | Week 08 | 118 | 0.5470 | 0.3268 |  |  |  |  |  |
|  |  |  |  | Week 16 | 118 | 0.5288 | 0.3711 |  |  |  |  |  |
|  |  |  |  | Week 28 | 100 | 0.3633 | 0.4163 |  |  |  |  |  |
|  | | | | | | | | | | | | |
|  | No to Yes | 41 | 10.5610 | Week 04 | 41 | 0.5551 | 0.4005 | 0.1692 | 0.4695 | -0.7566 | 1.0950 | 0.7190 |
|  |  |  |  | Week 08 | 40 | 0.7453 | 0.4955 | 0.1984 | 0.5805 | -0.9461 | 1.3429 | 0.7329 |
|  |  |  |  | Week 16 | 41 | 1.3892 | 0.5713 | 0.8604 | 0.6697 | -0.4600 | 2.1807 | 0.2003 |
|  |  |  |  | Week 28 | 40 | 3.6659 | 0.6275 | 3.3025 | 0.7425 | 1.8380 | 4.7671 | <.0001 |
|  | | | | | | | | | | | | |
|  | Yes to Yes | 49 | 14.9728 | Week 04 | 49 | 0.6164 | 0.4096 | 0.2305 | 0.4817 | -0.7193 | 1.1802 | 0.6328 |
|  |  |  |  | Week 08 | 49 | 1.2483 | 0.4979 | 0.7014 | 0.5936 | -0.4691 | 1.8718 | 0.2388 |
|  |  |  |  | Week 16 | 49 | 1.0553 | 0.5728 | 0.5264 | 0.6876 | -0.8292 | 1.8821 | 0.4448 |
|  |  |  |  | Week 28 | 47 | 1.4672 | 0.6287 | 1.1038 | 0.7629 | -0.4010 | 2.6086 | 0.1496 |
|  | | | | | | | | | | | | |
| QLS01 | No at week 28 | 118 | 3.6889 | Week 04 | 118 | -0.1880 | 0.0898 |  |  |  |  |  |
|  |  |  |  | Week 08 | 118 | -0.1022 | 0.0965 |  |  |  |  |  |
|  |  |  |  | Week 16 | 118 | -0.0135 | 0.1026 |  |  |  |  |  |
|  |  |  |  | Week 28 | 100 | 0.1438 | 0.1146 |  |  |  |  |  |
|  | | | | | | | | | | | | |
|  | No to Yes | 41 | 4.0697 | Week 04 | 41 | 0.1723 | 0.1333 | 0.3602 | 0.1555 | 0.0535 | 0.6669 | 0.0216 |
|  |  |  |  | Week 08 | 40 | 0.1582 | 0.1466 | 0.2603 | 0.1709 | -0.0766 | 0.5973 | 0.1292 |
|  |  |  |  | Week 16 | 41 | 0.3913 | 0.1571 | 0.4048 | 0.1833 | 0.0434 | 0.7663 | 0.0283 |
|  |  |  |  | Week 28 | 40 | 0.5073 | 0.1723 | 0.3635 | 0.2030 | -0.0370 | 0.7639 | 0.0750 |
|  | | | | | | | | | | | | |
|  | Yes to Yes | 49 | 4.7172 | Week 04 | 49 | 0.2641 | 0.1309 | 0.4521 | 0.1514 | 0.1536 | 0.7506 | 0.0032 |
|  |  |  |  | Week 08 | 49 | 0.3374 | 0.1423 | 0.4395 | 0.1656 | 0.1131 | 0.7660 | 0.0086 |
|  |  |  |  | Week 16 | 49 | 0.5812 | 0.1526 | 0.5947 | 0.1784 | 0.2430 | 0.9463 | 0.0010 |
|  |  |  |  | Week 28 | 47 | 0.7328 | 0.1669 | 0.5890 | 0.1980 | 0.1985 | 0.9794 | 0.0033 |
|  | | | | | | | | | | | | |
| QLS02 | No at week 28 | 118 | 2.8220 | Week 04 | 118 | 0.0475 | 0.1116 |  |  |  |  |  |
|  |  |  |  | Week 08 | 118 | 0.1857 | 0.1102 |  |  |  |  |  |
|  |  |  |  | Week 16 | 118 | 0.2197 | 0.1229 |  |  |  |  |  |
|  |  |  |  | Week 28 | 100 | 0.0822 | 0.1307 |  |  |  |  |  |
|  | | | | | | | | | | | | |
|  | No to Yes | 41 | 2.7561 | Week 04 | 41 | 0.3455 | 0.1704 | 0.2980 | 0.1979 | -0.0922 | 0.6883 | 0.1337 |
|  |  |  |  | Week 08 | 40 | 0.3057 | 0.1687 | 0.1200 | 0.1958 | -0.2659 | 0.5060 | 0.5405 |
|  |  |  |  | Week 16 | 41 | 0.5684 | 0.1914 | 0.3487 | 0.2223 | -0.0895 | 0.7869 | 0.1182 |
|  |  |  |  | Week 28 | 40 | 0.6917 | 0.1961 | 0.6096 | 0.2306 | 0.1547 | 1.0645 | 0.0089 |
|  | | | | | | | | | | | | |
|  | Yes to Yes | 49 | 3.4490 | Week 04 | 49 | 0.1659 | 0.1630 | 0.1184 | 0.1874 | -0.2512 | 0.4879 | 0.5284 |
|  |  |  |  | Week 08 | 49 | 0.5540 | 0.1607 | 0.3684 | 0.1845 | 0.0045 | 0.7322 | 0.0472 |
|  |  |  |  | Week 16 | 49 | 0.5835 | 0.1817 | 0.3638 | 0.2105 | -0.0512 | 0.7788 | 0.0854 |
|  |  |  |  | Week 28 | 47 | 0.7793 | 0.1868 | 0.6971 | 0.2191 | 0.2650 | 1.1293 | 0.0017 |
|  | | | | | | | | | | | | |
| QLS03 | No at week 28 | 118 | 2.0169 | Week 04 | 118 | 0.1647 | 0.1164 |  |  |  |  |  |
|  |  |  |  | Week 08 | 118 | 0.3971 | 0.1211 |  |  |  |  |  |
|  |  |  |  | Week 16 | 118 | 0.2398 | 0.1402 |  |  |  |  |  |
|  |  |  |  | Week 28 | 100 | 0.2434 | 0.1537 |  |  |  |  |  |
|  | | | | | | | | | | | | |
|  | No to Yes | 41 | 1.9512 | Week 04 | 41 | 0.3601 | 0.1751 | 0.1954 | 0.2030 | -0.2049 | 0.5957 | 0.3369 |
|  |  |  |  | Week 08 | 40 | 0.5733 | 0.1852 | 0.1762 | 0.2145 | -0.2467 | 0.5990 | 0.4125 |
|  |  |  |  | Week 16 | 41 | 0.8355 | 0.2195 | 0.5957 | 0.2543 | 0.0943 | 1.0971 | 0.0201 |
|  |  |  |  | Week 28 | 40 | 0.9976 | 0.2350 | 0.7542 | 0.2749 | 0.2119 | 1.2964 | 0.0067 |
|  | | | | | | | | | | | | |
|  | Yes to Yes | 49 | 3.0204 | Week 04 | 49 | 0.2908 | 0.1692 | 0.1262 | 0.1954 | -0.2591 | 0.5114 | 0.5192 |
|  |  |  |  | Week 08 | 49 | 0.4907 | 0.1773 | 0.0936 | 0.2054 | -0.3114 | 0.4985 | 0.6492 |
|  |  |  |  | Week 16 | 49 | 0.4046 | 0.2096 | 0.1648 | 0.2450 | -0.3182 | 0.6479 | 0.5018 |
|  |  |  |  | Week 28 | 47 | 0.6676 | 0.2245 | 0.4242 | 0.2654 | -0.0993 | 0.9477 | 0.1116 |
|  | | | | | | | | | | | | |
| QLS04 | No at week 28 | 118 | 2.4322 | Week 04 | 118 | 0.0141 | 0.1058 |  |  |  |  |  |
|  |  |  |  | Week 08 | 118 | 0.1723 | 0.1103 |  |  |  |  |  |
|  |  |  |  | Week 16 | 118 | 0.1243 | 0.1211 |  |  |  |  |  |
|  |  |  |  | Week 28 | 100 | 0.2585 | 0.1259 |  |  |  |  |  |
|  | | | | | | | | | | | | |
|  | No to Yes | 41 | 2.5854 | Week 04 | 41 | 0.1158 | 0.1597 | 0.1017 | 0.1858 | -0.2646 | 0.4679 | 0.5847 |
|  |  |  |  | Week 08 | 40 | 0.2040 | 0.1693 | 0.0317 | 0.1966 | -0.3560 | 0.4194 | 0.8720 |
|  |  |  |  | Week 16 | 41 | 0.4702 | 0.1881 | 0.3459 | 0.2187 | -0.0852 | 0.7770 | 0.1152 |
|  |  |  |  | Week 28 | 40 | 0.8140 | 0.1894 | 0.5556 | 0.2224 | 0.1168 | 0.9943 | 0.0134 |
|  | | | | | | | | | | | | |
|  | Yes to Yes | 49 | 3.2449 | Week 04 | 49 | 0.4415 | 0.1538 | 0.4274 | 0.1776 | 0.0771 | 0.7776 | 0.0170 |
|  |  |  |  | Week 08 | 49 | 0.5100 | 0.1614 | 0.3377 | 0.1870 | -0.0310 | 0.7064 | 0.0724 |
|  |  |  |  | Week 16 | 49 | 0.7101 | 0.1794 | 0.5858 | 0.2092 | 0.1733 | 0.9983 | 0.0056 |
|  |  |  |  | Week 28 | 47 | 0.9479 | 0.1814 | 0.6895 | 0.2133 | 0.2686 | 1.1103 | 0.0015 |
|  | | | | | | | | | | | | |
| QLS05 | No at week 28 | 118 | 2.7797 | Week 04 | 118 | 0.1196 | 0.0805 |  |  |  |  |  |
|  |  |  |  | Week 08 | 118 | 0.1529 | 0.0978 |  |  |  |  |  |
|  |  |  |  | Week 16 | 118 | 0.2894 | 0.1073 |  |  |  |  |  |
|  |  |  |  | Week 28 | 100 | 0.2367 | 0.1144 |  |  |  |  |  |
|  | | | | | | | | | | | | |
|  | No to Yes | 41 | 3.1463 | Week 04 | 41 | 0.0916 | 0.1195 | -0.0280 | 0.1394 | -0.3028 | 0.2468 | 0.8410 |
|  |  |  |  | Week 08 | 40 | 0.2350 | 0.1526 | 0.0821 | 0.1777 | -0.2682 | 0.4325 | 0.6444 |
|  |  |  |  | Week 16 | 41 | 0.2732 | 0.1685 | -0.0162 | 0.1965 | -0.4036 | 0.3712 | 0.9344 |
|  |  |  |  | Week 28 | 40 | 0.7354 | 0.1739 | 0.4987 | 0.2050 | 0.0944 | 0.9030 | 0.0159 |
|  | | | | | | | | | | | | |
|  | Yes to Yes | 49 | 3.7143 | Week 04 | 49 | 0.2764 | 0.1169 | 0.1568 | 0.1349 | -0.1091 | 0.4228 | 0.2463 |
|  |  |  |  | Week 08 | 49 | 0.5937 | 0.1458 | 0.4408 | 0.1709 | 0.1040 | 0.7777 | 0.0106 |
|  |  |  |  | Week 16 | 49 | 0.6477 | 0.1613 | 0.3584 | 0.1900 | -0.0162 | 0.7329 | 0.0607 |
|  |  |  |  | Week 28 | 47 | 0.7703 | 0.1670 | 0.5336 | 0.1987 | 0.1419 | 0.9253 | 0.0078 |
|  | | | | | | | | | | | | |
| QLS06 | No at week 28 | 118 | 2.3136 | Week 04 | 118 | 0.0486 | 0.0889 |  |  |  |  |  |
|  |  |  |  | Week 08 | 118 | 0.1329 | 0.0937 |  |  |  |  |  |
|  |  |  |  | Week 16 | 118 | 0.1068 | 0.1168 |  |  |  |  |  |
|  |  |  |  | Week 28 | 100 | 0.1931 | 0.1210 |  |  |  |  |  |
|  | | | | | | | | | | | | |
|  | No to Yes | 41 | 2.4390 | Week 04 | 41 | 0.1182 | 0.1329 | 0.0695 | 0.1544 | -0.2349 | 0.3740 | 0.6529 |
|  |  |  |  | Week 08 | 40 | 0.2148 | 0.1427 | 0.0820 | 0.1656 | -0.2445 | 0.4084 | 0.6212 |
|  |  |  |  | Week 16 | 41 | 0.5128 | 0.1839 | 0.4060 | 0.2135 | -0.0150 | 0.8270 | 0.0587 |
|  |  |  |  | Week 28 | 40 | 1.0118 | 0.1850 | 0.8187 | 0.2167 | 0.3915 | 1.2460 | 0.0002 |
|  | | | | | | | | | | | | |
|  | Yes to Yes | 49 | 3.4082 | Week 04 | 49 | 0.5027 | 0.1307 | 0.4541 | 0.1508 | 0.1567 | 0.7514 | 0.0029 |
|  |  |  |  | Week 08 | 49 | 0.6498 | 0.1387 | 0.5169 | 0.1609 | 0.1996 | 0.8341 | 0.0015 |
|  |  |  |  | Week 16 | 49 | 0.7824 | 0.1775 | 0.6756 | 0.2088 | 0.2639 | 1.0874 | 0.0014 |
|  |  |  |  | Week 28 | 47 | 0.6833 | 0.1791 | 0.4902 | 0.2120 | 0.0721 | 0.9083 | 0.0218 |
|  | | | | | | | | | | | | |
| QLS07 | No at week 28 | 118 | 3.0000 | Week 04 | 118 | 0.0506 | 0.0871 |  |  |  |  |  |
|  |  |  |  | Week 08 | 118 | 0.1244 | 0.0941 |  |  |  |  |  |
|  |  |  |  | Week 16 | 118 | 0.0219 | 0.1065 |  |  |  |  |  |
|  |  |  |  | Week 28 | 100 | 0.0728 | 0.1205 |  |  |  |  |  |
|  | | | | | | | | | | | | |
|  | No to Yes | 41 | 3.2927 | Week 04 | 41 | 0.0209 | 0.1303 | -0.0296 | 0.1520 | -0.3293 | 0.2700 | 0.8456 |
|  |  |  |  | Week 08 | 40 | 0.1196 | 0.1443 | -0.0048 | 0.1681 | -0.3361 | 0.3266 | 0.9773 |
|  |  |  |  | Week 16 | 41 | 0.3997 | 0.1656 | 0.3778 | 0.1931 | -0.0029 | 0.7584 | 0.0517 |
|  |  |  |  | Week 28 | 40 | 0.6389 | 0.1811 | 0.5661 | 0.2139 | 0.1443 | 0.9879 | 0.0088 |
|  | | | | | | | | | | | | |
|  | Yes to Yes | 49 | 4.1633 | Week 04 | 49 | 0.3334 | 0.1278 | 0.2829 | 0.1507 | -0.0142 | 0.5799 | 0.0619 |
|  |  |  |  | Week 08 | 49 | 0.5230 | 0.1398 | 0.3987 | 0.1655 | 0.0723 | 0.7250 | 0.0169 |
|  |  |  |  | Week 16 | 49 | 0.6059 | 0.1608 | 0.5840 | 0.1914 | 0.2066 | 0.9614 | 0.0026 |
|  |  |  |  | Week 28 | 47 | 0.6896 | 0.1775 | 0.6168 | 0.2140 | 0.1948 | 1.0389 | 0.0044 |
|  | | | | | | | | | | | | |
| QLS08 | No at week 28 | 118 | 2.1695 | Week 04 | 118 | -0.0271 | 0.0951 |  |  |  |  |  |
|  |  |  |  | Week 08 | 118 | 0.1170 | 0.1008 |  |  |  |  |  |
|  |  |  |  | Week 16 | 118 | 0.1263 | 0.1177 |  |  |  |  |  |
|  |  |  |  | Week 28 | 100 | 0.0525 | 0.1455 |  |  |  |  |  |
|  | | | | | | | | | | | | |
|  | No to Yes | 41 | 2.4878 | Week 04 | 41 | 0.1110 | 0.1411 | 0.1381 | 0.1643 | -0.1859 | 0.4621 | 0.4017 |
|  |  |  |  | Week 08 | 40 | 0.2024 | 0.1528 | 0.0854 | 0.1777 | -0.2649 | 0.4356 | 0.6313 |
|  |  |  |  | Week 16 | 41 | 0.3828 | 0.1833 | 0.2565 | 0.2133 | -0.1641 | 0.6771 | 0.2306 |
|  |  |  |  | Week 28 | 40 | 0.4380 | 0.2235 | 0.3856 | 0.2630 | -0.1331 | 0.9042 | 0.1442 |
|  | | | | | | | | | | | | |
|  | Yes to Yes | 49 | 3.0816 | Week 04 | 49 | 0.3690 | 0.1368 | 0.3961 | 0.1571 | 0.0863 | 0.7060 | 0.0125 |
|  |  |  |  | Week 08 | 49 | 0.2873 | 0.1464 | 0.1703 | 0.1691 | -0.1631 | 0.5037 | 0.3152 |
|  |  |  |  | Week 16 | 49 | 0.3887 | 0.1746 | 0.2624 | 0.2039 | -0.1396 | 0.6644 | 0.1996 |
|  |  |  |  | Week 28 | 47 | 0.3953 | 0.2118 | 0.3428 | 0.2524 | -0.1550 | 0.8407 | 0.1760 |
|  | | | | | | | | | | | | |
| QLS09 | No at week 28 | 118 | 1.8898 | Week 04 | 118 | 0.0311 | 0.1033 |  |  |  |  |  |
|  |  |  |  | Week 08 | 118 | -0.0130 | 0.1100 |  |  |  |  |  |
|  |  |  |  | Week 16 | 118 | 0.0103 | 0.1217 |  |  |  |  |  |
|  |  |  |  | Week 28 | 100 | -0.0037 | 0.1368 |  |  |  |  |  |
|  | | | | | | | | | | | | |
|  | No to Yes | 41 | 2.4146 | Week 04 | 41 | 0.0395 | 0.1538 | 0.0084 | 0.1796 | -0.3458 | 0.3625 | 0.9628 |
|  |  |  |  | Week 08 | 40 | 0.2552 | 0.1666 | 0.2682 | 0.1945 | -0.1153 | 0.6517 | 0.1694 |
|  |  |  |  | Week 16 | 41 | 0.5743 | 0.1867 | 0.5640 | 0.2181 | 0.1340 | 0.9940 | 0.0104 |
|  |  |  |  | Week 28 | 40 | 0.9383 | 0.2054 | 0.9419 | 0.2425 | 0.4637 | 1.4202 | 0.0001 |
|  | | | | | | | | | | | | |
|  | Yes to Yes | 49 | 3.8571 | Week 04 | 49 | 0.2018 | 0.1573 | 0.1708 | 0.1842 | -0.1924 | 0.5340 | 0.3550 |
|  |  |  |  | Week 08 | 49 | 0.4107 | 0.1688 | 0.4237 | 0.1988 | 0.0318 | 0.8157 | 0.0343 |
|  |  |  |  | Week 16 | 49 | 0.2524 | 0.1887 | 0.2422 | 0.2239 | -0.1992 | 0.6835 | 0.2806 |
|  |  |  |  | Week 28 | 47 | 0.1728 | 0.2076 | 0.1765 | 0.2499 | -0.3163 | 0.6692 | 0.4809 |
|  | | | | | | | | | | | | |
| QLS10 | No at week 28 | 118 | 2.1610 | Week 04 | 118 | 0.1639 | 0.0908 |  |  |  |  |  |
|  |  |  |  | Week 08 | 118 | 0.1704 | 0.1011 |  |  |  |  |  |
|  |  |  |  | Week 16 | 118 | 0.1673 | 0.1130 |  |  |  |  |  |
|  |  |  |  | Week 28 | 100 | 0.0653 | 0.1242 |  |  |  |  |  |
|  | | | | | | | | | | | | |
|  | No to Yes | 41 | 2.4878 | Week 04 | 41 | 0.2930 | 0.1342 | 0.1291 | 0.1564 | -0.1792 | 0.4374 | 0.4100 |
|  |  |  |  | Week 08 | 40 | 0.4939 | 0.1541 | 0.3235 | 0.1795 | -0.0303 | 0.6774 | 0.0729 |
|  |  |  |  | Week 16 | 41 | 0.6279 | 0.1749 | 0.4606 | 0.2037 | 0.0589 | 0.8622 | 0.0248 |
|  |  |  |  | Week 28 | 40 | 1.0013 | 0.1863 | 0.9360 | 0.2196 | 0.5028 | 1.3691 | <.0001 |
|  | | | | | | | | | | | | |
|  | Yes to Yes | 49 | 3.4286 | Week 04 | 49 | 0.3479 | 0.1349 | 0.1840 | 0.1552 | -0.1221 | 0.4901 | 0.2372 |
|  |  |  |  | Week 08 | 49 | 0.5186 | 0.1524 | 0.3482 | 0.1774 | -0.0016 | 0.6980 | 0.0511 |
|  |  |  |  | Week 16 | 49 | 0.6507 | 0.1723 | 0.4834 | 0.2022 | 0.0847 | 0.8821 | 0.0177 |
|  |  |  |  | Week 28 | 47 | 0.8035 | 0.1834 | 0.7382 | 0.2186 | 0.3071 | 1.1693 | 0.0009 |
|  | | | | | | | | | | | | |
| QLS11 | No at week 28 | 118 | 2.3305 | Week 04 | 118 | 0.0478 | 0.0987 |  |  |  |  |  |
|  |  |  |  | Week 08 | 118 | 0.0444 | 0.1139 |  |  |  |  |  |
|  |  |  |  | Week 16 | 118 | -0.0056 | 0.1267 |  |  |  |  |  |
|  |  |  |  | Week 28 | 100 | 0.0176 | 0.1337 |  |  |  |  |  |
|  | | | | | | | | | | | | |
|  | No to Yes | 41 | 2.8537 | Week 04 | 41 | 0.1592 | 0.1441 | 0.1114 | 0.1688 | -0.2214 | 0.4441 | 0.5100 |
|  |  |  |  | Week 08 | 40 | 0.0010 | 0.1732 | -0.0434 | 0.2024 | -0.4425 | 0.3557 | 0.8305 |
|  |  |  |  | Week 16 | 41 | 0.1142 | 0.1954 | 0.1198 | 0.2285 | -0.3307 | 0.5704 | 0.6006 |
|  |  |  |  | Week 28 | 40 | 0.7868 | 0.2008 | 0.7693 | 0.2370 | 0.3018 | 1.2368 | 0.0014 |
|  | | | | | | | | | | | | |
|  | Yes to Yes | 49 | 3.9184 | Week 04 | 49 | 0.2688 | 0.1416 | 0.2210 | 0.1667 | -0.1077 | 0.5497 | 0.1865 |
|  |  |  |  | Week 08 | 49 | 0.4146 | 0.1678 | 0.3702 | 0.1991 | -0.0224 | 0.7629 | 0.0645 |
|  |  |  |  | Week 16 | 49 | 0.3043 | 0.1894 | 0.3099 | 0.2258 | -0.1353 | 0.7551 | 0.1714 |
|  |  |  |  | Week 28 | 47 | 0.3870 | 0.1949 | 0.3695 | 0.2338 | -0.0918 | 0.8307 | 0.1158 |
|  | | | | | | | | | | | | |
| QLS12 | No at week 28 | 118 | 2.1582 | Week 04 | 118 | -0.0552 | 0.0920 |  |  |  |  |  |
|  |  |  |  | Week 08 | 118 | 0.1043 | 0.1066 |  |  |  |  |  |
|  |  |  |  | Week 16 | 118 | 0.0962 | 0.1200 |  |  |  |  |  |
|  |  |  |  | Week 28 | 100 | 0.0637 | 0.1292 |  |  |  |  |  |
|  | | | | | | | | | | | | |
|  | No to Yes | 41 | 2.8049 | Week 04 | 41 | 0.0646 | 0.1335 | 0.1197 | 0.1570 | -0.1899 | 0.4294 | 0.4467 |
|  |  |  |  | Week 08 | 40 | -0.0044 | 0.1610 | -0.1087 | 0.1892 | -0.4818 | 0.2643 | 0.5663 |
|  |  |  |  | Week 16 | 41 | 0.0748 | 0.1840 | -0.0214 | 0.2163 | -0.4479 | 0.4052 | 0.9214 |
|  |  |  |  | Week 28 | 40 | 0.9409 | 0.1941 | 0.8773 | 0.2302 | 0.4232 | 1.3313 | 0.0002 |
|  | | | | | | | | | | | | |
|  | Yes to Yes | 49 | 3.7687 | Week 04 | 49 | 0.1483 | 0.1341 | 0.2034 | 0.1582 | -0.1085 | 0.5154 | 0.2000 |
|  |  |  |  | Week 08 | 49 | 0.3558 | 0.1592 | 0.2515 | 0.1898 | -0.1228 | 0.6258 | 0.1868 |
|  |  |  |  | Week 16 | 49 | 0.3433 | 0.1817 | 0.2472 | 0.2181 | -0.1828 | 0.6771 | 0.2583 |
|  |  |  |  | Week 28 | 47 | 0.5290 | 0.1922 | 0.4653 | 0.2327 | 0.0063 | 0.9243 | 0.0469 |
|  | | | | | | | | | | | | |
| QLS13 | No at week 28 | 118 | 2.0763 | Week 04 | 118 | 0.0471 | 0.1012 |  |  |  |  |  |
|  |  |  |  | Week 08 | 118 | 0.0796 | 0.1027 |  |  |  |  |  |
|  |  |  |  | Week 16 | 118 | 0.0323 | 0.1182 |  |  |  |  |  |
|  |  |  |  | Week 28 | 100 | -0.0139 | 0.1245 |  |  |  |  |  |
|  | | | | | | | | | | | | |
|  | No to Yes | 41 | 2.7805 | Week 04 | 41 | 0.3746 | 0.1518 | 0.3275 | 0.1790 | -0.0254 | 0.6805 | 0.0688 |
|  |  |  |  | Week 08 | 40 | 0.4715 | 0.1555 | 0.3918 | 0.1830 | 0.0310 | 0.7527 | 0.0334 |
|  |  |  |  | Week 16 | 41 | 0.4849 | 0.1824 | 0.4526 | 0.2150 | 0.0287 | 0.8766 | 0.0365 |
|  |  |  |  | Week 28 | 40 | 0.7331 | 0.1847 | 0.7470 | 0.2203 | 0.3124 | 1.1816 | 0.0008 |
|  | | | | | | | | | | | | |
|  | Yes to Yes | 49 | 3.3673 | Week 04 | 49 | 0.3178 | 0.1493 | 0.2707 | 0.1766 | -0.0776 | 0.6189 | 0.1269 |
|  |  |  |  | Week 08 | 49 | 0.5266 | 0.1517 | 0.4470 | 0.1797 | 0.0927 | 0.8013 | 0.0137 |
|  |  |  |  | Week 16 | 49 | 0.6038 | 0.1776 | 0.5715 | 0.2121 | 0.1534 | 0.9897 | 0.0076 |
|  |  |  |  | Week 28 | 47 | 0.7510 | 0.1811 | 0.7648 | 0.2187 | 0.3334 | 1.1962 | 0.0006 |
|  | | | | | | | | | | | | |
| QLS14 | No at week 28 | 118 | 2.4153 | Week 04 | 118 | 0.0355 | 0.0849 |  |  |  |  |  |
|  |  |  |  | Week 08 | 118 | 0.0733 | 0.0951 |  |  |  |  |  |
|  |  |  |  | Week 16 | 118 | 0.0294 | 0.1039 |  |  |  |  |  |
|  |  |  |  | Week 28 | 100 | 0.0903 | 0.1190 |  |  |  |  |  |
|  | | | | | | | | | | | | |
|  | No to Yes | 41 | 3.2439 | Week 04 | 41 | 0.3369 | 0.1253 | 0.3014 | 0.1489 | 0.0077 | 0.5951 | 0.0443 |
|  |  |  |  | Week 08 | 40 | 0.2587 | 0.1444 | 0.1854 | 0.1713 | -0.1523 | 0.5232 | 0.2803 |
|  |  |  |  | Week 16 | 41 | 0.3864 | 0.1588 | 0.3570 | 0.1888 | -0.0153 | 0.7293 | 0.0601 |
|  |  |  |  | Week 28 | 40 | 0.6882 | 0.1751 | 0.5979 | 0.2114 | 0.1808 | 1.0149 | 0.0052 |
|  | | | | | | | | | | | | |
|  | Yes to Yes | 49 | 3.9184 | Week 04 | 49 | 0.4106 | 0.1264 | 0.3751 | 0.1511 | 0.0772 | 0.6730 | 0.0138 |
|  |  |  |  | Week 08 | 49 | 0.5472 | 0.1434 | 0.4739 | 0.1728 | 0.1332 | 0.8146 | 0.0066 |
|  |  |  |  | Week 16 | 49 | 0.6462 | 0.1580 | 0.6168 | 0.1913 | 0.2397 | 0.9939 | 0.0015 |
|  |  |  |  | Week 28 | 47 | 0.7300 | 0.1758 | 0.6397 | 0.2170 | 0.2117 | 1.0677 | 0.0036 |
|  | | | | | | | | | | | | |
| QLS15 | No at week 28 | 118 | 2.6441 | Week 04 | 118 | -0.0286 | 0.0996 |  |  |  |  |  |
|  |  |  |  | Week 08 | 118 | 0.0070 | 0.1072 |  |  |  |  |  |
|  |  |  |  | Week 16 | 118 | 0.0739 | 0.1184 |  |  |  |  |  |
|  |  |  |  | Week 28 | 100 | 0.0242 | 0.1262 |  |  |  |  |  |
|  | | | | | | | | | | | | |
|  | No to Yes | 41 | 3.0244 | Week 04 | 41 | 0.3948 | 0.1503 | 0.4234 | 0.1753 | 0.0778 | 0.7690 | 0.0166 |
|  |  |  |  | Week 08 | 40 | 0.2868 | 0.1655 | 0.2798 | 0.1928 | -0.1003 | 0.6599 | 0.1482 |
|  |  |  |  | Week 16 | 41 | 0.3372 | 0.1848 | 0.2633 | 0.2156 | -0.1617 | 0.6883 | 0.2233 |
|  |  |  |  | Week 28 | 40 | 0.3809 | 0.1906 | 0.3567 | 0.2249 | -0.0868 | 0.8002 | 0.1143 |
|  | | | | | | | | | | | | |
|  | Yes to Yes | 49 | 3.5102 | Week 04 | 49 | 0.4857 | 0.1452 | 0.5143 | 0.1685 | 0.1821 | 0.8464 | 0.0026 |
|  |  |  |  | Week 08 | 49 | 0.5362 | 0.1579 | 0.5293 | 0.1842 | 0.1662 | 0.8924 | 0.0045 |
|  |  |  |  | Week 16 | 49 | 0.5334 | 0.1765 | 0.4595 | 0.2071 | 0.0512 | 0.8678 | 0.0276 |
|  |  |  |  | Week 28 | 47 | 0.4465 | 0.1827 | 0.4223 | 0.2173 | -0.0063 | 0.8509 | 0.0534 |
|  | | | | | | | | | | | | |
| QLS16 | No at week 28 | 118 | 2.9492 | Week 04 | 118 | 0.0350 | 0.0957 |  |  |  |  |  |
|  |  |  |  | Week 08 | 118 | 0.0978 | 0.1072 |  |  |  |  |  |
|  |  |  |  | Week 16 | 118 | 0.0069 | 0.1171 |  |  |  |  |  |
|  |  |  |  | Week 28 | 100 | 0.1241 | 0.1255 |  |  |  |  |  |
|  | | | | | | | | | | | | |
|  | No to Yes | 41 | 3.4146 | Week 04 | 41 | 0.0866 | 0.1421 | 0.0515 | 0.1664 | -0.2765 | 0.3796 | 0.7570 |
|  |  |  |  | Week 08 | 40 | 0.1659 | 0.1647 | 0.0681 | 0.1926 | -0.3116 | 0.4477 | 0.7240 |
|  |  |  |  | Week 16 | 41 | 0.3559 | 0.1814 | 0.3490 | 0.2123 | -0.0697 | 0.7676 | 0.1018 |
|  |  |  |  | Week 28 | 40 | 0.6616 | 0.1885 | 0.5375 | 0.2229 | 0.0979 | 0.9771 | 0.0168 |
|  | | | | | | | | | | | | |
|  | Yes to Yes | 49 | 3.9592 | Week 04 | 49 | 0.6232 | 0.1379 | 0.5882 | 0.1611 | 0.2705 | 0.9059 | 0.0003 |
|  |  |  |  | Week 08 | 49 | 0.7720 | 0.1573 | 0.6742 | 0.1853 | 0.3089 | 1.0395 | 0.0003 |
|  |  |  |  | Week 16 | 49 | 0.7812 | 0.1738 | 0.7743 | 0.2056 | 0.3689 | 1.1797 | 0.0002 |
|  |  |  |  | Week 28 | 47 | 1.0135 | 0.1814 | 0.8894 | 0.2167 | 0.4620 | 1.3168 | <.0001 |
|  | | | | | | | | | | | | |
| QLS17 | No at week 28 | 118 | 2.8814 | Week 04 | 118 | 0.0212 | 0.1088 |  |  |  |  |  |
|  |  |  |  | Week 08 | 118 | -0.0033 | 0.1245 |  |  |  |  |  |
|  |  |  |  | Week 16 | 118 | 0.0069 | 0.1358 |  |  |  |  |  |
|  |  |  |  | Week 28 | 100 | -0.0924 | 0.1421 |  |  |  |  |  |
|  | | | | | | | | | | | | |
|  | No to Yes | 41 | 3.1463 | Week 04 | 41 | 0.2101 | 0.1600 | 0.1888 | 0.1862 | -0.1784 | 0.5560 | 0.3119 |
|  |  |  |  | Week 08 | 40 | 0.2421 | 0.1903 | 0.2454 | 0.2210 | -0.1904 | 0.6811 | 0.2682 |
|  |  |  |  | Week 16 | 41 | 0.6266 | 0.2096 | 0.6198 | 0.2438 | 0.1391 | 1.1004 | 0.0118 |
|  |  |  |  | Week 28 | 40 | 0.7850 | 0.2120 | 0.8773 | 0.2490 | 0.3862 | 1.3685 | 0.0005 |
|  | | | | | | | | | | | | |
|  | Yes to Yes | 49 | 4.5306 | Week 04 | 49 | 0.4406 | 0.1605 | 0.4194 | 0.1879 | 0.0489 | 0.7899 | 0.0267 |
|  |  |  |  | Week 08 | 49 | 0.5552 | 0.1881 | 0.5585 | 0.2223 | 0.1202 | 0.9969 | 0.0128 |
|  |  |  |  | Week 16 | 49 | 0.4749 | 0.2076 | 0.4681 | 0.2465 | -0.0179 | 0.9540 | 0.0590 |
|  |  |  |  | Week 28 | 47 | 0.6326 | 0.2107 | 0.7249 | 0.2530 | 0.2259 | 1.2240 | 0.0046 |
|  | | | | | | | | | | | | |
| QLS18 | No at week 28 | 118 | 4.0169 | Week 04 | 118 | 0.0957 | 0.0722 |  |  |  |  |  |
|  |  |  |  | Week 08 | 118 | 0.0438 | 0.0837 |  |  |  |  |  |
|  |  |  |  | Week 16 | 118 | 0.1256 | 0.0847 |  |  |  |  |  |
|  |  |  |  | Week 28 | 100 | 0.2111 | 0.0989 |  |  |  |  |  |
|  | | | | | | | | | | | | |
|  | No to Yes | 41 | 4.4634 | Week 04 | 41 | 0.1670 | 0.1058 | 0.0713 | 0.1242 | -0.1737 | 0.3162 | 0.5668 |
|  |  |  |  | Week 08 | 40 | 0.2993 | 0.1282 | 0.2554 | 0.1501 | -0.0405 | 0.5514 | 0.0904 |
|  |  |  |  | Week 16 | 41 | 0.3739 | 0.1292 | 0.2483 | 0.1514 | -0.0503 | 0.5469 | 0.1026 |
|  |  |  |  | Week 28 | 40 | 0.3342 | 0.1486 | 0.1231 | 0.1761 | -0.2242 | 0.4704 | 0.4853 |
|  | | | | | | | | | | | | |
|  | Yes to Yes | 49 | 5.0408 | Week 04 | 49 | 0.2753 | 0.1028 | 0.1796 | 0.1208 | -0.0586 | 0.4179 | 0.1387 |
|  |  |  |  | Week 08 | 49 | 0.2725 | 0.1225 | 0.2286 | 0.1452 | -0.0577 | 0.5149 | 0.1170 |
|  |  |  |  | Week 16 | 49 | 0.3404 | 0.1242 | 0.2148 | 0.1473 | -0.0756 | 0.5052 | 0.1463 |
|  |  |  |  | Week 28 | 47 | 0.2994 | 0.1427 | 0.0883 | 0.1722 | -0.2513 | 0.4279 | 0.6087 |
|  | | | | | | | | | | | | |
| QLS19 | No at week 28 | 118 | 2.9661 | Week 04 | 118 | -0.1612 | 0.0912 |  |  |  |  |  |
|  |  |  |  | Week 08 | 118 | -0.0419 | 0.0958 |  |  |  |  |  |
|  |  |  |  | Week 16 | 118 | 0.0255 | 0.1064 |  |  |  |  |  |
|  |  |  |  | Week 28 | 100 | -0.0312 | 0.1024 |  |  |  |  |  |
|  | | | | | | | | | | | | |
|  | No to Yes | 41 | 3.0732 | Week 04 | 41 | 0.1627 | 0.1380 | 0.3240 | 0.1604 | 0.0077 | 0.6402 | 0.0447 |
|  |  |  |  | Week 08 | 40 | 0.3431 | 0.1477 | 0.3849 | 0.1714 | 0.0469 | 0.7229 | 0.0258 |
|  |  |  |  | Week 16 | 41 | 0.1213 | 0.1660 | 0.0959 | 0.1928 | -0.2843 | 0.4761 | 0.6195 |
|  |  |  |  | Week 28 | 40 | 0.3573 | 0.1519 | 0.3885 | 0.1785 | 0.0365 | 0.7405 | 0.0307 |
|  | | | | | | | | | | | | |
|  | Yes to Yes | 49 | 3.8367 | Week 04 | 49 | 0.2276 | 0.1353 | 0.3889 | 0.1564 | 0.0806 | 0.6972 | 0.0137 |
|  |  |  |  | Week 08 | 49 | 0.3735 | 0.1431 | 0.4153 | 0.1661 | 0.0879 | 0.7428 | 0.0132 |
|  |  |  |  | Week 16 | 49 | 0.3292 | 0.1609 | 0.3037 | 0.1880 | -0.0670 | 0.6745 | 0.1078 |
|  |  |  |  | Week 28 | 47 | 0.6085 | 0.1484 | 0.6397 | 0.1750 | 0.2946 | 0.9849 | 0.0003 |
|  | | | | | | | | | | | | |
| QLS20 | No at week 28 | 118 | 3.3644 | Week 04 | 117 | 0.0182 | 0.0709 |  |  |  |  |  |
|  |  |  |  | Week 08 | 118 | 0.0827 | 0.0717 |  |  |  |  |  |
|  |  |  |  | Week 16 | 118 | 0.0240 | 0.0856 |  |  |  |  |  |
|  |  |  |  | Week 28 | 100 | -0.0288 | 0.0930 |  |  |  |  |  |
|  | | | | | | | | | | | | |
|  | No to Yes | 41 | 3.8049 | Week 04 | 41 | 0.2593 | 0.1063 | 0.2411 | 0.1247 | -0.0047 | 0.4869 | 0.0545 |
|  |  |  |  | Week 08 | 40 | 0.3219 | 0.1094 | 0.2392 | 0.1279 | -0.0129 | 0.4913 | 0.0628 |
|  |  |  |  | Week 16 | 41 | 0.4211 | 0.1336 | 0.3971 | 0.1564 | 0.0888 | 0.7054 | 0.0118 |
|  |  |  |  | Week 28 | 40 | 0.4700 | 0.1398 | 0.4988 | 0.1658 | 0.1716 | 0.8260 | 0.0030 |
|  | | | | | | | | | | | | |
|  | Yes to Yes | 49 | 4.4286 | Week 04 | 49 | 0.1854 | 0.1038 | 0.1672 | 0.1224 | -0.0741 | 0.4085 | 0.1734 |
|  |  |  |  | Week 08 | 49 | 0.3625 | 0.1057 | 0.2798 | 0.1245 | 0.0344 | 0.5253 | 0.0257 |
|  |  |  |  | Week 16 | 49 | 0.2937 | 0.1289 | 0.2697 | 0.1532 | -0.0323 | 0.5717 | 0.0798 |
|  |  |  |  | Week 28 | 47 | 0.4718 | 0.1356 | 0.5006 | 0.1633 | 0.1784 | 0.8229 | 0.0025 |
|  | | | | | | | | | | | | |
| QLS21 | No at week 28 | 118 | 3.9407 | Week 04 | 118 | 0.0091 | 0.0853 |  |  |  |  |  |
|  |  |  |  | Week 08 | 118 | 0.0632 | 0.0898 |  |  |  |  |  |
|  |  |  |  | Week 16 | 118 | -0.0032 | 0.1056 |  |  |  |  |  |
|  |  |  |  | Week 28 | 100 | 0.0057 | 0.1077 |  |  |  |  |  |
|  | | | | | | | | | | | | |
|  | No to Yes | 41 | 4.5610 | Week 04 | 41 | 0.2682 | 0.1265 | 0.2591 | 0.1492 | -0.0351 | 0.5533 | 0.0840 |
|  |  |  |  | Week 08 | 40 | 0.3367 | 0.1357 | 0.2735 | 0.1598 | -0.0415 | 0.5884 | 0.0885 |
|  |  |  |  | Week 16 | 41 | 0.4550 | 0.1633 | 0.4582 | 0.1924 | 0.0789 | 0.8375 | 0.0182 |
|  |  |  |  | Week 28 | 40 | 0.4839 | 0.1606 | 0.4782 | 0.1912 | 0.1010 | 0.8554 | 0.0132 |
|  | | | | | | | | | | | | |
|  | Yes to Yes | 49 | 5.0612 | Week 04 | 49 | 0.0463 | 0.1230 | 0.0372 | 0.1450 | -0.2488 | 0.3233 | 0.7976 |
|  |  |  |  | Week 08 | 49 | 0.2029 | 0.1306 | 0.1397 | 0.1546 | -0.1651 | 0.4445 | 0.3672 |
|  |  |  |  | Week 16 | 49 | 0.2894 | 0.1570 | 0.2926 | 0.1874 | -0.0769 | 0.6620 | 0.1200 |
|  |  |  |  | Week 28 | 47 | 0.3160 | 0.1550 | 0.3103 | 0.1868 | -0.0582 | 0.6788 | 0.0984 |
|  | | | | | | | | | | | | |
| TOTAL SCORE | No at week 28 | 118 | 57.0165 | Week 04 | 118 | 1.5650 | 0.8088 |  |  |  |  |  |
|  |  |  |  | Week 08 | 118 | 3.1866 | 0.9937 |  |  |  |  |  |
|  |  |  |  | Week 16 | 118 | 2.9050 | 1.2857 |  |  |  |  |  |
|  |  |  |  | Week 28 | 100 | 2.6905 | 1.4410 |  |  |  |  |  |
|  | | | | | | | | | | | | |
|  | No to Yes | 41 | 64.8014 | Week 04 | 41 | 4.0978 | 1.1602 | 2.5329 | 1.3640 | -0.1567 | 5.2224 | 0.0648 |
|  |  |  |  | Week 08 | 40 | 5.3884 | 1.5008 | 2.2018 | 1.7626 | -1.2739 | 5.6775 | 0.2131 |
|  |  |  |  | Week 16 | 41 | 8.6313 | 1.9975 | 5.7262 | 2.3479 | 1.0969 | 10.3555 | 0.0156 |
|  |  |  |  | Week 28 | 40 | 14.2819 | 2.1926 | 11.5914 | 2.5969 | 6.4692 | 16.7136 | <.0001 |
|  | | | | | | | | | | | | |
|  | Yes to Yes | 49 | 81.4247 | Week 04 | 49 | 4.6189 | 1.2089 | 3.0539 | 1.4283 | 0.2375 | 5.8703 | 0.0337 |
|  |  |  |  | Week 08 | 49 | 7.3475 | 1.5309 | 4.1609 | 1.8386 | 0.5351 | 7.7867 | 0.0247 |
|  |  |  |  | Week 16 | 49 | 8.3613 | 2.0263 | 5.4563 | 2.4609 | 0.6040 | 10.3085 | 0.0277 |
|  |  |  |  | Week 28 | 47 | 10.5451 | 2.2262 | 7.8546 | 2.7302 | 2.4695 | 13.2397 | 0.0045 |
|  | | | | | | | | | | | | |
| Unstructured for covariance matrix  Visit, age group, region and interaction between shift group and visit as factors and interaction between baseline QLS and visit as covariate  Scores for item 1/12 equal to 9 has been changed according to the SAP | | | | | | | | | | | | |
